# Effect of Different Local Antibiotic Regimens on Prevention of Postoperative Infection in Clean Surgical Wounds: A Systematic Review and Network Meta-analysis

**DOI:** 10.1097/ASW.0000000000000094

**Published:** 2024-02-09

**Authors:** Hai Bang Pan, Yan Cui, Zhi Hang Wu, Ying Meng, Tian Ming Wang, Qi Fu, Qian Chen, Quan Xin Chen, Bo Wang

**Affiliations:** At First Clinical Medical College, Gansu University of Chinese Medicine, Lanzhou City, Gansu Province, China, Hai Bang Pan, MD, is Associate Professor, and Yan Cui, MM, and Zhi Hang Wu, MM, are Graduate Students in Surgery. Ying Meng, MM, is Primary Pharmacist, Zibo City, Shandong Province, China. Also at the First Clinical Medical College, Gansu University of Chinese Medicine, Tian Ming Wang, MM; Qi Fu, MM; Qian Chen, MM; and Quan Xin Chen, MM, are Graduate Students in Surgery. Bo Wang, MM, is Associate Professor, School of Nursing, Gansu University of Traditional Chinese Medicine.

**Keywords:** antibiotic, clean wound, network meta-analysis, prevention, surgical site infection, surgical wound, topical

## Abstract

**OBJECTIVE:**

To compare the efficacy of several local antibiotic regimens in preventing surgical site infection (SSI) in clean surgical wounds.

**DATA SOURCES:**

The authors searched CNKI (China National Knowledge Infrastructure), the VIP (VIP information resource integration service platform), Wanfang Data knowledge service platform (WANFANG), SinoMed, Cochrane Library, EMBASE, and PubMed.

**STUDY SELECTION:**

A total of 20 randomized controlled trials published between January 1, 2000 and April 1, 2021 were included in this meta-analysis.

**DATA EXTRACTION:**

Authors extracted the name of the first author, publication date, country, type of surgery, follow-up time, mean age of participants, sample size of each group, interventions, outcome indicators, and study type from each article.

**DATA SYNTHESIS:**

The overall effectiveness of eight local managements in reducing the incidence of the SSI effect were compared through the SUCRA (surface under the cumulative ranking curve) probabilities. The results of a network meta-analysis demonstrated that gentamicin ointment (odds ratio [OR], 0.16; 95% CI, 0.04–0.60), mupirocin ointment (OR, 0.44; 95% CI, 0.21–0.94), and gentamicin soaking of the graft (OR, 0.63; 95% CI, 0.44–0.91) significantly reduced the incidence of SSI compared with control. Further, vancomycin soaking of the graft (86.7%) ranked first, followed by gentamicin ointment (81.1%), gentamicin irrigation (79.9%), mupirocin ointment (56.8%), triple antibiotic ointment (47.8%), gentamicin soaking of the graft (42.3%), and vancomycin powder (22.1%); ampicillin powder (17.8%) was the least effective drug.

**CONCLUSIONS:**

The findings indicate that local antibiotics combined with conventional antibiotics in the wound before wound closure are effective in reducing the incidence of SSI in clean surgical wounds. Vancomycin inoculation of the graft exhibited the best effect.

## INTRODUCTION

Surgical site infection (SSI) is one of the main causes of healthcare-associated infections, accounting for 15% of all such infections.^1^ An SSI is a postoperative infection of deep tissues and organs near the surgical incision, usually within 30 days after surgery.^2^ Once infection occurs, the patient’s morbidity, mortality, length of hospital stay, and economic burden all increase, and quality of life decreases.^3^ Risk factors such as uncontrolled diabetes, advanced age, smoking, immunosuppression, inadequate disinfection, inappropriate use of antibiotics, and a long course of disease all contribute to SSI development.^4^

With the increasing number of surgical procedures worldwide, the prevention of SSI is critical in all surgical stages.^4^ According to guidelines from the CDC on SSI prevention, the use of antimicrobial agents such as creams, solutions, or powders at surgical incisions is not recommended for SSI prevention, and high-quality evidence is lacking.^5^ However, SSIs can occur despite the administration of IV antibiotics because these can only reach tissues with an adequate blood supply. In contrast, topical antibiotic therapy can provide a higher concentration directly to the surgical site, potentially contributing to greater efficiency in preventing biofilm formation and fracture-related infections.^6^ At present, the primary rationale for reducing topical antibiotic usage is drug resistance. However, a considerable number of randomized controlled trials (RCTs) and meta-analyses have confirmed the effectiveness of local antibiotic use at the site of injury in reducing the incidence of SSI with no adverse drug reactions. Thus, local use of antibiotics has certain advantages over systemic use. Given the above contradiction, it is necessary to analyze and discuss this topic further.

Over the past 20 years, various topical drugs for preventing postoperative infections have reduced the incidence of SSI to varying degrees. Ushirozako et al^7^ reported that intrawound vancomycin powder (VP) lowered the risk of SSI after posterior spinal surgery by half without systemic adverse events. Iorio et al^8^ suggested that topical use of VP plus povidone-iodine not only reduced the incidence of SSI but also saved money. Similarly, Wong et al^9^ found that applying mupirocin at the catheter outlet was safe and decreased wound infection rates. A retrospective cohort study comparing the incidence of SSI after conventional treatment and gentamicin-collagen sponge prophylaxis in patients undergoing mastectomy showed that the application of gentamicin-collagen sponges reduced the incidence of SSI.^10^ However, because different medicines have different effects, the optimal drug regimen remains unclear.

Traditional pairwise comparison meta-analysis can only distinguish which is best between two comparators. In contrast, network meta-analysis (NMA) informs decision-making by assessing the relative effectiveness of more than two alternative interventions for the same disease.^11^ Thus, with NMA, it is possible to select the best drug for a certain disease and provide more direct evidence for clinical use. In the present study, the authors used NMA based on the frequentist framework to compare the effectiveness of different intraoperative and postoperative local antibiotics in preventing SSI and provide evidence-based evidence for clinical drug selection. The NMA was registered with the PROSPERO database (CRD42022331452).

## METHODS

### Search Strategy

The authors searched the CNKI (China National Knowledge Infrastructure), VIP (VIP information resource integration service platform), WANFANG (Wanfang Data knowledge service platform), CBM, Cochrane Library, EMBASE, and PubMed databases using the search terms “surgical wound infection,” “vancomycin,” “mupirocin,” “gentamicin,” “load antibiotic,” “topical antibiotics,” and free words for each keyword. Different retrieval modes were combined for retrieval according to the retrieval modes of different databases, such as ((((“vancomycin”[MeSH]) OR “gentamicin”[MeSH]) OR “Mupirocin”[MeSH]) OR ((load antibiotic) OR (topical antibiotics))) AND (“surgical wound infection”[MeSH]). The search was limited to articles published between January 1, 2000 and January 1, 2022; the language was not restricted. The authors also searched the references in the initial meta-analysis literature and review articles for further relevant studies.

### Article Selection

Inclusion and exclusion criteria were determined prior to retrieval. Original RCTs satisfying the following criteria were included: (1) patients who underwent surgery, not restricted by age or sex; (2) patients only used antibiotic locally during or after surgery; (3) the observation group and control group only received intraoperative or postoperative topical drugs, including vancomycin, gentamicin, mupirocin, other local antibiotics, or a combination thereof; and (4) postoperative SSI rates were reported. Literature with the following characteristics was excluded: (1) patients with preoperative infection, (2) contamination surgery, (3) preoperative medication (not excluding prophylactic perioperative IV antibiotics), (4) animal experiments, (5) lack of a control, (6) reviews, (7) case reports, (8) data or full text not available, and (9) patents.

Two researchers screened all the titles and abstracts of the retrieved literature. After preliminary exclusion and inclusion of the literature, the full texts were downloaded for further screening. Any disagreements were settled with a third researcher to determine the final articles included in the meta-analysis.

### Data Extraction

Two researchers analyzed all relevant material from the retrieved articles separately and utilized data extraction tables particularly prepared for this review. Any disagreements between the two reviewers were settled by consulting with a third researcher to determine the final inclusion criterion. The first author’s name, publication date, country, type of surgery, follow-up time, mean age, sample size of each group, interventions, outcome indicators, and study type were extracted from the literature.

### Quality Assessment

The researchers used the Cochrane Collaboration tool to assess the risk of bias in the RCTs, including random sequence generation, allocation concealment, participant and personnel blinding, outcome assessment blinding, incomplete outcome data, selective reporting, and other biases. The visualization was achieved using the RevMan software (version 5.4.1; The Cochrane Collaboration, 2020).

### Statistical Analysis

Stata software (version 15; StataCorp) was used to draw network evidence maps of different local drugs for visually representing the quantity of evidence and comparing relationships among different drugs. First, researchers analyzed direct comparisons among different topical agents. They performed an NMA using the frequentist framework random-effects model, which allowed indirect and direct evidence to be combined in the analysis and ranked the efficacy of different drugs. The inconsistency test was first performed when test results were *P* > .05, which implied no statistical significance and indicated consistency. A consistency model was used for NMA. The surface under the cumulative ranking curve (SUCRA) probabilities were used to rank treatment methods for each outcome; greater SUCRA probabilities in each simulation suggest a better possibility of being the optimal treatment regimen. Finally, a funnel plot was created to depict publication bias for all available treatments. *P* < .05 was considered significant.

## RESULTS

### Identification of Relevant Studies

**Figure 1.**
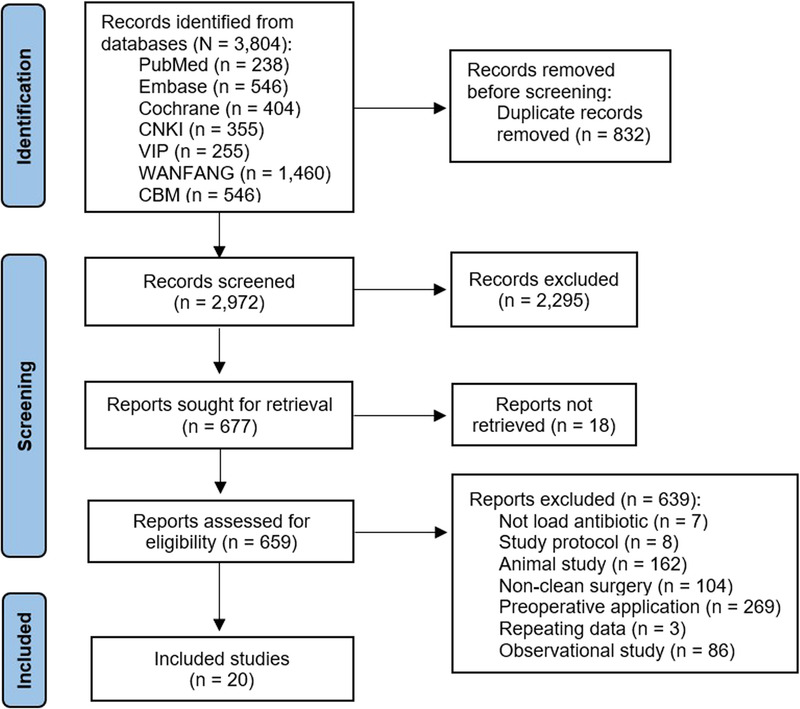
STUDY SELECTION FLOW DIAGRAM

A total of 3,804 articles were initially retrieved from database searches; of these, 832 were duplicates. The titles and abstracts of the remaining 2,972 articles were reviewed to exclude review articles, patents, animal experiments, and intervention mismatches. Following this screening, the researchers read the full text of the remaining 677 articles. A total of 657 studies were excluded (ie, inconsistent indicators, abstracts, inability to obtain the full text, preoperative or systemic medication, small sample trials, observational studies, and no-control trials). Thus, 20 articles were included in the NMA. The literature retrieval and screening process is illustrated in Figure 1.

### Characteristics of the Included Trials

**Figure 2.**
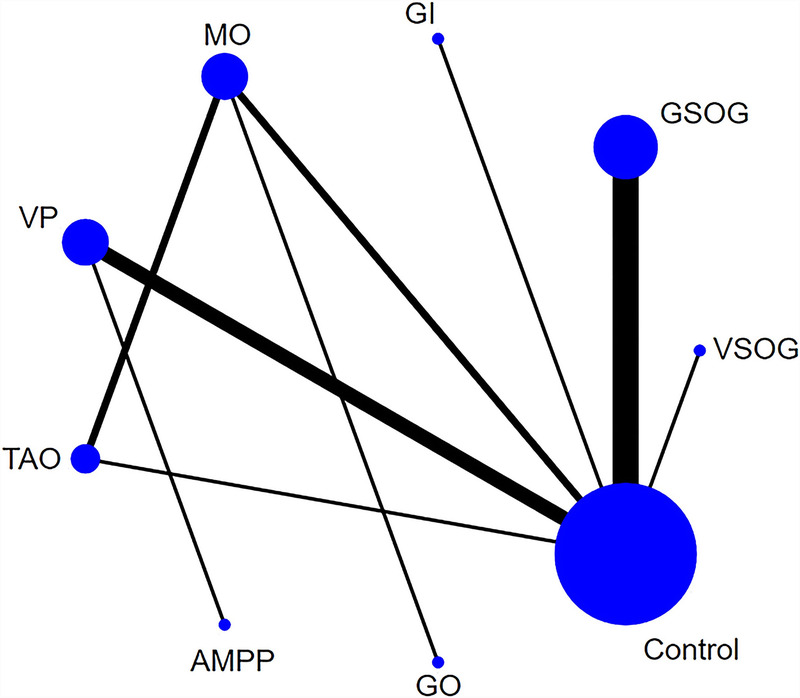
NETWORK EVIDENCE MAP OF DIFFERENT LOCAL DRUGS Abbreviations: AMPP, ampicillin powder; GI, gentamicin irrigation; GO, gentamicin ointment; GSOG, gentamicin soaking of the graft; MO, mupirocin ointment; TAO, triple antibiotic ointment; VP, vancomycin powder; VSOG, vancomycin soaking of the graft.

The 20 RCTs included in the meta-analysis include a variety of surgical fields, such as spinal, joint, cardiac, and implant surgery.^6,12–30^ Eight different interventions were evaluated: VP, vancomycin soaking of the graft (VSOG), gentamicin ointment (GO), gentamicin irrigation (GI), gentamicin soaking of the graft (GSOG), mupirocin ointment (MO), ampicillin powder, and triple antibiotic ointment. Control groups included patients receiving preoperative prophylactic IV antibiotics and/or placebos. Other basic information is presented in Table 1. The network evidence diagram of the treatment measures is shown in Figure 2.

**Table 1.** BASIC CHARACTERISTICS OF THE INCLUDED RCTs

Authors, Year, Country	Type of Surgery	Intervention		Follow-up, mo	n		Age, Mean (SD), y	
		Treatment 1	Treatment 2		Group 1	Group 2	Group 1	Group 2
O’Toole et al,^6^ 2021, USA	Tibial plateau or pilon fracture	VP	Control	12	481	499	45.4 (14.0)	46.1 (13.5)
Chiu and Lin,^12^ 2009, China	Knee arthroplasty	VSOG	Control	90 ± 3	93	90	71 (8.4)	70 (7.8)
McQuillan et al,^13^ 2012, Canada	Peritoneal dialysis	MO	TAO	18	100	101	61.02 (13.66)	59.36 (15.04)
Mirzashahi et al,^14^ 2018, Italy	Spinal	VP	Control	NR	193	187	NR	NR
Takeuchi et al,^15^ 2018, Japan	Spinal	VP	AMPP	12	116	114	66 (14)	68 (14)
Bernardini et al,^16^ 2005, USA	Peritoneal dialysis	MO	GO	5	66	67	51 (15)	54 (15)
Bennett-Guerrero et al,^17^ 2010, USA	Cardiac	GSOG	Control	>3	753	749	64.2	64.9
Dixon et al,^18^ 2006, Australia	Excision of skin lesions	MO	Control	6	262	269	59.1	60.5
Eklund et al,^19^ 2005, Finland	Cardiac	GSOG	Control	3	272	270	64.4 (9.3)	64.7 (9.3)
Friberg et al,^20^ 2005, Sweden	Cardiac	GSOG	Control	2	983	967	68	68
Schimmer et al,^21^ 2017, Germany	Cardiac	GSOG	Control	3	336	332	67.7 (9.6)	67.8 (9.7)
Schimmer et al,^22^ 2012, USA	Heart	GSOG	Control	>1	353	367	69	69
Westberg et al,^23^ 2015, Norway	Hip arthroplasty	GSOG	Control	>6	329	355	82.0 (7.6)	83 (8.5)
Wong et al,^9^ 2003, China	Peritoneal dialysis	MO	Control	>5	78	88	60 (12)	59 (13)
Wübbeke et al,^24^ 2020, the Netherlands	Vascular	GSOG	Control	1	151	137	69 (9.2)	70 (10.4)
He et al,^25^ 2015, China	Craniotomy hematoma removal	GI	Control	0.25	42	42	63.31 (15.67)	61.27 (10.42)
Lok et al et al,^26^ 2023, USA	Hemodialysis	TAO	Control	>6	83	79	68	64
Hood et al,^27^ 2004, USA	Soft tissue wound	MO	TAO	0.25	50	49	21.6 (14.9)	27.7 (19.9)
Salimi et al,^28^ 2022, Iran	Spinal	VP	Control	3	187	188	51.7	52.3
Ludwig do Nascimento et al,^29^ 2019, Brazil	Spinal	VP	Control	3	48	48	43 (14.88)	43 (14.88)

Abbreviations: AMPP, ampicillin powder; GI, gentamicin irrigation; GO, gentamicin ointment; GSOG, gentamicin soaking of the graft; MO, mupirocin ointment; NR, not reported; RCT, randomized controlled trial; TAO, triple antibiotic ointment; VP, vancomycin powder; VSOG, vancomycin soaking of the graft.

### Quality Assessment

**Figure 3.**
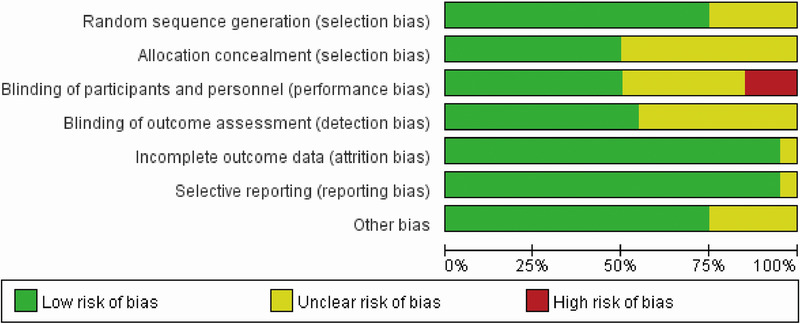
QUALITY EVALUATION CHART OF RANDOMIZED CONTROLLED TRIALS

Figure 3 shows the results of the RCT quality evaluation. The highest risk of bias in the included RCTs related to blinding (performance bias). Some literature could not blind surgeons because of surgical reasons. The overall quality of the literature was acceptable.

### Network Meta-analysis

The 20 included RCTs included 9,974 participants and evaluated eight topical antibiotic regimens and one control. When the inconsistent model was examined, it showed significant results (*P* = .0247). Thus, the inconsistent model was used to analyze this batch of data rather than the consistent model. The analysis results suggested that GO (odds ratio [OR], 0.16; 95% CI, 0.04–0.60), MO (OR, 0.44; 95% CI, 0.21–0.94), and GSOG (OR, 0.63; 95% CI, 0.44–0.91) significantly reduced the incidence of SSI compared with the control. Further, GO was more effective in preventing postoperative SSI than MO (OR, 0.18; 95% CI, 0.04–0.74). Detailed results are listed in Table 2.

**Table 2.** RESULTS OF THE NETWORK META-ANALYSIS ON SSI RATE

VSOG	2.73 (0.11, 69.42)	1.68 (0.02, 117.72)	7.41 (0.35, 158.11)	8.89 (0.40, 194.99)	10.51 (0.53, 208.72)	15.31 (0.75, 314.42)	23.58 (0.65, 861.80)	16.67 (0.86, 323.68)
0.37 (0.01, 9.33)	**GO**	0.61 (0.02, 16.82)	2.72 (0.95, 7.80)	3.26 (0.83, 12.75)	3.85 (1.00, 14.79)	5.61 (1.35, 23.30)	8.64 (0.77, 96.93)	6.11 (1.67, 22.31)
0.60 (0.01, 41.84)	1.63 (0.06, 44.50)	**GI**	4.42 (0.19, 101.75)	5.30 (0.22, 125.40)	6.26 (0.29, 134.54)	9.13 (0.41, 202.51)	14.06 (0.36, 548.39)	9.94 (0.47, 208.76)
0.13 (0.01, 2.88)	0.37 (0.13, 1.06)	0.23 (0.01, 5.21)	**MO**	1.20 (0.50, 2.85)	1.42 (0.61, 3.27)	2.06 (0.79, 5.38)	3.18 (0.36, 28.00)	2.25 (1.06, 4.77)
0.11 (0.01, 2.47)	0.31 (0.08, 1.20)	0.19 (0.01, 4.47)	0.83 (0.35, 1.98)	**TAO**	1.18 (0.46, 3.01)	1.72 (0.61, 4.84)	2.65 (0.29, 24.18)	1.88 (0.79, 4.44)
0.10 (0.00, 1.89)	0.26 (0.07, 1.00)	0.16 (0.01, 3.43)	0.71 (0.31, 1.63)	0.85 (0.33, 2.16)	**GSOG**	1.46 (0.73, 2.90)	2.24 (0.28, 17.79)	1.59 (1.10, 2.29)
0.07 (0.00, 1.34)	0.18 (0.04, 0.74)^a^	0.11 (0.00, 2.43)	0.48 (0.19, 1.26)	0.58 (0.21, 1.63)	0.69 (0.35, 1.36)	**VP**	1.54 (0.22, 10.86)	1.09 (0.61, 1.94)
0.04 (0.00, 1.55)	0.12 (0.01, 1.30)	0.07 (0.00, 2.78)	0.31 (0.04, 2.77)	0.38 (0.04, 3.43)	0.45 (0.06, 3.53)	0.65 (0.09, 4.58)	**AMPP**	0.71 (0.09, 5.42)
0.06 (0.00, 1.17)	0.16 (0.04, 0.60)^a^	0.10 (0.00, 2.11)	0.44 (0.21, 0.94)^a^	0.53 (0.23, 1.26)	0.63 (0.44, 0.91)^a^	0.92 (0.51, 1.64)	1.41 (0.18, 10.85)	**Control**

Abbreviations: AMPP, ampicillin powder; GI, gentamicin irrigation; GO, gentamicin ointment; GSOG, gentamicin soaking of the graft; MO, mupirocin ointment; SSI, surgical site infection; TAO, triple antibiotic ointment; VP, vancomycin powder; VSOG, vancomycin soaking of the graft.

^a^Statistically significant.

**Figure 4.**
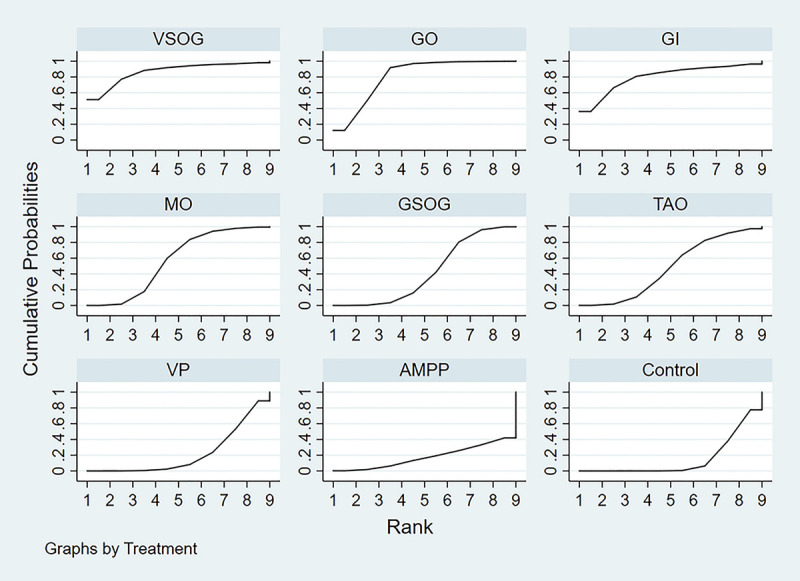
A PLOT OF THE SURFACE UNDER THE CUMULATIVE RANKING CURVES Abbreviations: AMPP, ampicillin powder; GI, gentamicin irrigation; GO, gentamicin ointment; GSOG, gentamicin soaking of the graft; MO, mupirocin ointment; TAO, triple antibiotic ointment; VP, vancomycin powder; VSOG, vancomycin soaking of the graft.

According to the SUCRA value, VSOG (86.7%) ranked first, followed by GO (81.1%), GI (79.9%), MO (56.8%), triple antibiotic ointment (47.8%), GSOG (42.3%), VP (22.1%), and ampicillin powder (17.8%). Control (15.3%) was the least effective. The ranking is illustrated as an area graph under the curve (Figure 4).

### Publication Bias

**Figure 5.**
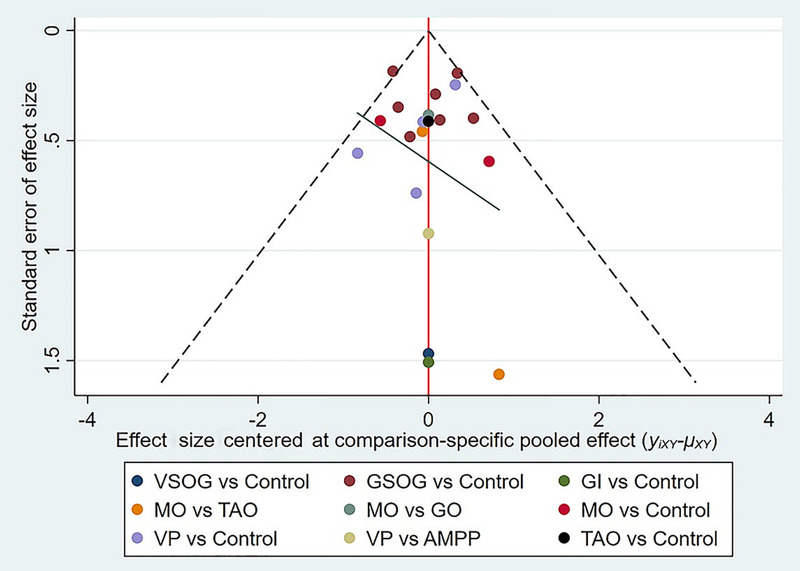
INVERTED FUNNEL DIAGRAM OF PUBLICATION BIAS EVALUATION Abbreviations: AMPP, ampicillin powder; GI, gentamicin irrigation; GO, gentamicin ointment; GSOG, gentamicin soaking of the graft; MO, mupirocin ointment; TAO, triple antibiotic ointment; VP, vancomycin powder; VSOG, vancomycin soaking of the graft.

A funnel plot (Figure 5) exhibits the publication bias results. There was asymmetry on both sides of the funnel, indicating a possibility of publication bias.

### Loop Inconsistency

**Figure 6.**
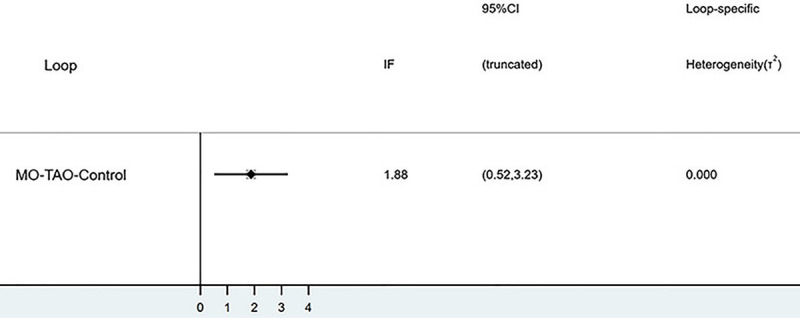
RESULTS OF THE LOOP INCONSISTENCY TEST Abbreviations: MO, mupirocin ointment; TAO, triple antibiotic ointment.

Figure 6 displays the outcomes of the loop consistency test. The contradiction is not significant, and the results have some credibility with an inconsistency factor of 1.88.

## DISCUSSION

Surgical site infections are a concern for all surgical patients, regardless of the procedure type or location.^30^ Preventive medication is essential for lowering SSI risk, but there is inconsistent evidence in the literature regarding the efficacy of various local antibiotics for this purpose. Further, the role of different local antibiotics in patients after aseptic operations has yet to be defined.

Statistical pooling of this NMA of 20 RCTs comparing eight local antibiotics revealed that VSOG is the most effective local antibiotic for SSI prevention. The strategies discussed herein are used in the context of routine antibiotic use. The effect of local antibiotics combined with conventional antibiotics in preventing postoperative infection is better than that of conventional antibiotics alone. In addition, the researchers also found that the local therapeutic effect of VSOG is better than GO, GSOG, and VP.

Most studies focus on characterizing certain types of surgery; however, the present study included multiple surgical procedures. As a result, it is difficult to compare these findings with published reports. However, there are similar studies, despite different study premises, which may assist in interpreting the results. A meta-analysis by Lee et al^31^ suggests there may be a significant risk reduction in SSI after mucosal head and neck surgery when local antibiotics are added to standard systemic prophylaxis. Another meta-analysis of randomized and quasi-randomized clinical trials of topical antibiotics after primary closure for the prevention of SSI suggested that topical antibiotics likely prevent SSI compared with no topical antibiotic or antiseptic.^32^ Although these studies have shown the effectiveness of topical antibiotics in preventing SSI, they do not indicate which is most effective.

Vancomycin, a glycopeptide antibiotic, is used to treat Gram-positive bacterial infections and is recommended by the Infectious Diseases Society of America as a first-line treatment against methicillin-resistant *Staphylococcus aureus* infections.^33^ Local use of VP in spinal surgery,^34^ skull surgery,^35^ and joint surgery^36^ has achieved positive results and lowered the incidence of SSI, with no adverse reactions. Renal function recovered within 1 week of vancomycin discontinuation in 44% to 75% of patients with nephrotoxicity.^37^ Adverse drug reactions to vancomycin were rarely mentioned in the RCTs included in the present review, confirming that a potential benefit of VP administration is a decreased likelihood of adverse drug reactions compared with systemic administration of vancomycin. Nevertheless, high-quality prospective studies are needed to confirm this result.

Vancomycin has been applied locally in multiple ways, including adding vancomycin to bone cement^38^ and soaking suturing tape and loop binding wire with vancomycin,^39^ among other new vehicles. Topical administration prevents rapid absorption of the drug and thus weakens its systemic effects.^36^ The present meta-analysis determined that VSOG was more effective than VP in preventing SSI. However, the appropriate dosage and pharmacokinetics of vancomycin applied to wounds have not yet been explored, and current dosages depend on the experience of the surgeon. A significant number of clinical and basic experiments are required to investigate the mechanism of action of vancomycin in local administration.

The results of this study also point to the efficacy of gentamicin in addition to vancomycin, particularly the second-ranked GI and third-ranked GO strategies. According to research by Wang et al,^40^ topical gentamicin use considerably shortens the time it takes for wounds to heal and boosts clinical efficacy in patients who have local wound infections or are at risk of infection.^39^ In a meta-analysis, Kowalewski et al^41^ confirmed the positive effect of gentamicin sponge. Gentamicin is particularly suitable for infections caused by Gram-negative pathogens and is effective against surface *Staphylococcus*. Topical gentamicin has several advantages: it does not damage renal function and has a low risk of drug-resistant pathogens, it kills bacteria by inhibiting protein synthesis, and it destroys the stability of the bacterial bilayer membrane.^24^ Wigmosta et al^42^ discovered that delivering gentamicin from the surface is superior to delivering gentamicin in a solution, which could improve the antimicrobial activity of orthopedic implants and lower the risk of infection-induced failure without reducing mammalian cell attachment. Further, gentamicin and curcumin-loaded lipid-polymer hybrid nanoparticles have excellent antibacterial properties and are promising antibacterial agents for the treatment of chronic infections and intracellular bacteria.^43^ In the present study, the meta-analysis determined that GSOG was less efficacious in preventing SSI than GO and GI, in contrast with vancomycin. This finding demonstrates that the change in dosage form has a substantial bearing on the effectiveness of various antibiotics.

In addition to the use of topical antibiotics, nonantibiotic therapies are also an effective means of preventing SSIs. Incisional negative-pressure wound therapy refers to the continuous or intermittent application of sub-atmospheric pressure to the wound dressing system. This accelerates primary wound healing by promoting the reduction of fluid accumulation in the wound, thereby reducing the risk of SSI.^44^ Meta-analyses by Norman et al^45^ and Zwanenburg et al^46^ both demonstrated a lower rate of SSI with negative-pressure wound therapy compared with standard dressings. There are also “biofilm busters” with peer-reviewed data, including HOCL (pure hypochlorous acid, pH 5.5),^47^ and the joint rinse experience including chlorhexidine, iodine, and olanexidine.^48^ Nonantibiotic therapy provides a new option for the prevention of wound infection and, when combined with topical antibiotics, may help reduce the selection pressure on microorganisms and thus reduce antibiotic resistance.

The present findings and those of numerous other studies support the notion that using topical antibiotics in conjunction with IV antibiotics can lessen the likelihood of adverse drug reactions and the development of resistant pathogens while also delivering higher local drug concentrations than systemic medications, effectively killing bacteria and preventing infection. The NMA in the present study revealed that the use of VP, VSOG, and GI combined with conventional antibiotics may be effective in preventing SSI; however, higher-quality RCTs are needed for validation. Of note, the present results only represent the effect of clean surgery. For nonclean surgery, the topical application of vancomycin and gentamicin may not have as much effect, especially for gastrointestinal surgery. In previous research, the topical use of gentamicin and vancomycin in appendectomy and colorectal surgery was not associated with a significant reduction in SSI.^49,50^

### Limitations

The studies included in this meta-analysis featured differing surgical locations, medication usage, and patient ages, all of which may have a bearing on the validity of the findings. To decrease the impact of small sample events, researchers removed studies with sample sizes under 50, and the articles included were of high quality and to some extent trustworthy. Further, factors such as inadequate nutrition, secondary surgical procedures, high body mass index, and preoperative lymphedema increase the risk of postoperative SSI development, whereas a preoperative lymphedema evaluation and micronutrients (25-hydroxyvitamin D) can positively impact SSI risk. However, the included articles did not support performing a subgroup analysis to investigate this issue.

## CONCLUSIONS

At present, IV injection before surgery and oral antibiotics after surgery are the preventive measures of choice for most surgeons, and few doctors add topical antibiotics to wound closure. However, topical antibiotics combined with oral antibiotics have advantages over oral antibiotics alone. The present meta-analysis confirmed that local antibiotics can reduce the incidence of adverse reactions and drug-resistant pathogens while achieving higher drug concentrations in the local area to kill bacteria and prevent infection compared with systemic drugs. Although the data suggest that vancomycin and gentamicin are both effective, the findings need to be further validated in randomized trials because of potential bias caused by variations in the doses used in different studies. In addition, overuse of antibiotics leads to increased resistance, and topical antibiotics combined with nonantibiotic treatment, which can reduce the antibiotic dose, may be a viable solution to address high resistance.
